# Repeated deep anterior lamellar keratoplasty combined with phacoemulsification

**DOI:** 10.3205/oc000252

**Published:** 2025-05-02

**Authors:** Mustafa Kayabaşı, Canan Asli Utine

**Affiliations:** 1 Department of Ophthalmology, Mus State Hospital, Muş, Turkey; 2 Department of Ophthalmology, Dokuz Eylul University, İzmir, Turkey; 3 İzmir Biomedicine and Genome Center, İzmir, Turkey

**Keywords:** corneal endothelium, deep anterior lamellar keratoplasty, phacoemulsification

## Abstract

**Objective:**

To report the clinical findings and results of a patient who underwent deep anterior lamellar keratoplasty (DALK) combined with phacoemulsification.

**Methods:**

Retrospective analysis of a case that underwent unsuccessful DALK surgery with no visual gain due to striations on the posterior surface of the donor, permanent interface irregularity, and scarring.

**Results:**

Two years after the first DALK surgery, a repeated DALK was performed in combination with phacoemulsification of the cataract that developed during this period. The graft was clear with no signs of rejection or endothelial decompensation, and corrected distance visual acuity was 6/10 in the postoperative last visit, one year after the surgery.

**Conclusions:**

Combining the DALK technique with phacoemulsification in patients with coexisting cataracts may help to achieve a good visual outcome and long-term graft survival. Even after an unsuccessful DALK experience, insisting on preserving the patient’s own endothelium resulted in successful vision restoration with no imposition of further risks for graft survival.

## Introduction

Deep anterior lamellar keratoplasty (DALK) has gained popularity in the past two decades. It is a valuable technique for patients with corneal stromal scarring associated with visual loss with an intact corneal endothelium [1], [2].

Herein, we report a case in which we performed two consecutive DALK surgeries, the second combined with phacoemulsification for the nuclear cataract.

## Case description

A 43-year-old female patient was treated for a year of fungal keratitis in the right eye before being admitted to Dokuz Eylul University, Department of Ophthalmology, Cornea Division. Upon the first admission, her corrected distance visual acuity (CDVA) in the Snellen lines was 2/10 on the right eye and 10/10 on the left eye. Stromal scarring was observed in the right cornea, which seemed to spare the endothelial layer. DALK surgery was performed in the right eye to achieve visual gain and preserve the patient’s endothelium. The big-bubble technique resulted in the exposure of smooth Descemet’s membrane of the host cornea. However, due to the inadequacy of the operating microscope during the surgery, stripping the Descemet membrane-endothelial complex of the donor cornea was uneasy. Despite prolonged surgery, the entire Descemet membrane-endothelial complex in the donor cornea could be removed with the help of a 7.25 mm diameter suction trephine, and the slightly deformed, 7.50 mm-diameter donor cornea was then transplanted over the recipient bed using 16 interrupted sutures since no backup graft tissue was present. Topical prednisolone and moxifloxacin eight times daily were given with a slow taper in the postoperative period in an effort to decrease the inflammation and haze formation at the graft-Descemet interface. Graft-bed apposition was rapidly achieved postoperatively with no double anterior chamber formation; however, CDVA remained at the level of 3/10 due to striations on the posterior surface of the donor, permanent interface irregularity, and scarring. 

During the follow-up, a nuclear cataract also developed in this eye, resulting in additional visual loss. Two years after the first surgery repeated DALK was performed in combination with phacoemulsification of the cataract. The video in Attachment 1 shows the combined surgical procedure in this case. The main corneal incision was created with a 2.4 mm blade. The anterior capsule was stained with trypan blue injected under an air bubble, followed by injection of a dispersive and a cohesive ophthalmic viscosurgical device (OVD) in soft shell technique, creating a continuous anterior capsulorhexis. After two side port incisions were made, a complete hydro-dissection was performed conventionally. Then, phacoemulsification of the nucleus was done using the phaco-chop nucleofractis technique. Complete cortical aspiration was performed by bimanual irrigation-aspiration. The capsular bag was inflated with a cohesive OVD, and a 23.00 diopter SA60AT (Alcon, Geneva, Switzerland) single-piece intraocular lens (IOL) was inserted in the bag. To avoid hyperopic postoperative refraction, the IOL power calculation was performed using the SRK/T formula, based on a postoperative estimated flat keratometry of 42.00 D. The remaining OVD were removed; the main incision and side ports were hydrated. During the phacoemulsification of the cataract, it was not easy to obtain a satisfactory visualization of the anterior chamber due to striations of the posterior surface of the donor cornea. We eliminated this challenge by using the retro-illumination technique with the operating microscope.

The remaining corneal interrupted sutures from the previous DALK surgery were removed. The graft-host junction was superficially separated with a stiletto knife to proceed with the second DALK procedure. The anterior corneal surface was trephined with a 7.50 mm diameter suction trephine, the same size as the first graft, set to a depth of about two-thirds of corneal thickness in the recipient’s eye. Extreme care was exerted to centralize the trephination over the existing graft to coincide with its borders. We ensured no fluid under the trephine, which might cause sliding and vacuum loss, leading to oblique trephination and an oval donor button with a resultant lack of graft-host apposition and undesirable high postoperative astigmatism. 

After trephination, the graft-host junction was gently dissected with a rounded blade. Air was then injected using a 30-gauge cannula to form a “big bubble” between the deep stroma and Descemet’s membrane and separate these layers. Since the main corneal incision for phacoemulsification was long enough to transverse the graft stroma, air bubble escape to the anterior chamber was noted. However, this did not imply a Descemet’s tear, and we continued DALK surgery as planned. The previous graft was cut into two halves by rounded corneal scissors, and Descemet’s membrane was separated and pushed backward by viscoelastic dissection. The air bubble in the anterior chamber guided us in better visualizing Descemet’s membrane. Following the total removal of the previous graft to expose the intact Descemet’s membrane, we prepared the new donor tissue. 

In the donor cornea, following trephination with a 7.75 mm punch, the endothelial surface of the graft was stained with trypan blue. The Descemet’s membrane-endothelium complex was removed by gently swabbing the posterior corneal surface in a centripetal direction with dry cellulose sponges and gentle dissection of the Descemet’s membrane using thin suture forceps. The graft was rinsed with a balanced salt solution to remove any remaining trypan blue dye. Later, the graft was sutured to the host using 16 interrupted 10/0 nylon sutures for a good graft-recipient apposition. The suture knots were buried, and the operation was terminated after an intracameral injection of 1 mg cefuroxime.

The patient was prescribed topical prednisolone and moxifloxacin eight times daily, with a slow taper in the postoperative period, followed by a four-day topical cyclosporine treatment. The patient was also prescribed topical moxifloxacin six times daily, which was tapered and ceased in 2 weeks upon complete re-epithelialization of the graft. Corneal epithelization and healing occurred quickly, without double anterior chamber formation or other complications. The sutures were extracted at the 6-month follow-up. At the last visit, 1 year postoperatively, CDVA was 0.6 in the right eye with a refraction of (–3.00 –2.50 @ 145). The graft was clear with no signs of rejection or anterior chamber reaction, and the IOL was centralized. The patient’s endothelial layer remained healthy during this process. Specular microscopy examination revealed an endothelial cell count of 1,779 cells/mm^2^. This decreased cell density was thought to result from fungal keratitis and accompanying intraocular inflammation. The magnitude of cell count was adequate to keep the stroma dry and transparent.

## Discussion

Lamellar keratoplasty is a selective tissue transplantation technique in which the diseased layers of the cornea are replaced, and the healthy layers of the cornea are retained. DALK is one of the anterior lamellar keratoplasty procedures that has gained popularity in the past two decades for eyes with stromal pathologies that do not affect the Descemet’s membrane and endothelium. Intact recipient Descemet membrane and endothelium are retained, and this procedure fully replaces the stroma [3]. A small amount of posterior stroma is left intact along with Descemet’s membrane in “pre-Descemetic DALK”, whereas in “Descemetic DALK”, all of the stroma is dissected from Descemet’s membrane by using the “big-bubble” technique, hydro dissection, viscodissection, manual dissection or by using femtosecond lasers [3], [4].

DALK can be performed in eyes with dystrophies that affect the corneal stroma and spare the endothelium-like lattice and granular corneal dystrophies; keratitis which healed with stromal scarring; ectasias like keratoconus without acute hydrops, pellucid marginal degeneration and keratoglobus [5]. This technique’s main advantage is minimizing penetrating keratoplasty (PK) complications, like immunologic rejection, and prolonging graft survival [1], [2]. In a retrospective study of 206 patients who underwent DALK or PK in a 1:1 ratio, Tan et al. [6] reported that 2-year graft survival was 98–100% in the DALK group, while it was 90% in the PK group. In another study published by Ahmad et al. [7], the 5-year graft survival rate for PK was found to be 76%, whereas a meta-analysis that evaluated 5-year graft survival after DALK reported survival rates of 1,970 patients and merged the graft survival rate as 90.4% [8].

Since the endothelium is protected, less immunosuppression is required in eyes with DALK in the postoperative period compared to PK. Accordingly, side effects of steroids will be seen less in patients with DALK [9]. Other advantages include being more trauma-resistant than full-thickness grafts, as in PK, since the recipient cornea is not incised in full thickness [2]. Although there is no significant difference in terms of postoperative CDVA, astigmatism is less common in patients with DALK since sutures can be removed earlier. Therefore, suture-related irritation and infections are less common [10]. Postoperative loss of endothelial cells in eyes with DALK is known to be less than in eyes with PK [1], [11]. Liu et al. [10] reported that the endothelial cell count in the postoperative 3^rd^ month was significantly higher after DALK than PK for necrotizing stromal keratitis. This favorable effect on endothelial cell count was even more prominent after cataract surgery [12].

Besides all these advantages, the DALK technique has disadvantages, just like any other technique. First of all, graft preparation is much more complicated than PK. Prolonged surgery time, surface irregularity at the graft-recipient bed interface leading to reduced visual gain, and the possibility of failure in graft-Descemet’s membrane attachment leading to the double anterior chamber in the early postoperative period are among the disadvantages of the DALK technique [1], [3], [13]. These challenges lead to a steep learning curve for DALK.

Patients who have advanced corneal scarring and coexisting cataracts also present surgical challenges. Phacoemulsification can be performed either before or after keratoplasty (sequential surgery) or simultaneously (combined surgery), depending on the severity of the cataract and the stage of the corneal pathology [14]. The combination of cataract surgery and corneal graft transplantation in the same session has several benefits over the two-staged procedure. Fewer follow-up visits, shorter visual rehabilitation time, and lower costs are the main benefits of the combined procedure [15]. Conventional “triple procedure” combines PK, cataract extraction, and IOL implantation. Phacoemulsification is the modern technique for cataract surgery and should be preferred over “open-sky” cataract extraction whenever corneal pathology allows adequate visualization of the anterior chamber. DALK surgery combined with phacoemulsification and IOL implantation is defined as a “modern triple procedure” in recent studies [16], [17]. No significant loss in endothelial cell density was found after phacoemulsification in eyes that developed cataracts after DALK, unlike those after PK [12], [18]. 

In a recent study, Alfonso-Bartolozzi et al. [14] reported the clinical outcomes of the triple procedure involving DALK, phacoemulsification, and IOL implantation in 43 eyes of 43 patients. They adjusted the sequence of procedures based on the level of corneal visibility. In 26 eyes with good corneal visibility, phacoemulsification was performed before stromal dissection, while 17 eyes with poor visibility underwent the DALK procedure to enhance clarity, enabling subsequent lensectomy. Eighty percent of patients experienced an improvement of 2 or more lines, and 70% achieved postoperative refraction within a ±2.0 D range. The authors concluded that the combined surgery of DALK, phacoemulsification, and IOL implantation is both effective and safe. They emphasized that surgical techniques can be modified according to corneal transparency to optimize efficacy and safety.

There are two main challenges when combining DALK and phacoemulsification. Firstly, performing phacoemulsification in an eye with a cloudy cornea might be difficult. Endo-illumination-assisted cataract surgery may help achieve better visualization of the anterior chamber. Secondly, there is an inherent disadvantage to the predictability of biometry measurements because of corneal power changes after keratoplasty [14], [16]. In our patient, anterior chamber visualization was complex due to striations on the posterior surface of the donor, interface irregularity, and scarring. We used retro illumination to visualize the anterior chamber better and injected trypan blue to visualize the anterior capsule better. The postoperative target refraction for our patient was –0.25 D, calculated with the SRK/T formula based on a postoperative estimated flat keratometry of 42.00 D; however, the postoperative refraction was (+3.25 –1.50 @ 175) in the 1^st^ month; (–3.00 –2.50 @ 145) in the 1^st^ year.

In conclusion, combining the DALK technique with phacoemulsification in patients with corneal stromal scarring and coexisting cataracts may help achieve a good visual outcome and long-term graft survival. Even in the presence of scarring and opacification in the graft-recipient interface, if the endothelium and Descemet’s membrane are intact, insisting on preserving the patient’s endothelium even after an unsuccessful lamellar keratoplasty can provide a good visual outcome with fewer complications than full-thickness grafts.

## Notes

### Video of surgical procedure in Attachment 1

The video in Attachment 1 shows the combined surgical procedure: Phacoemulsification with the phaco-chop technique was combined with repeated DALK surgery after a previous unsuccessful lamellar grafting. Visualization during phacoemulsification was improved by using retroillumination and trypan blue anterior capsular stain. The main corneal incision, long enough to transverse the former graft, led to air bubble escape into the anterior chamber during “big-bubble” formation. However, this did not impede a successful repeated DALK surgery.

### Ethics statement and patient consent

The study followed the tenets of the Declaration of Helsinki. The patient signed written informed consent for the research use of clinical records and data included in the study.

### Conference presentation

This study was presented as a poster at the Turkish Ophthalmology Society’s 55^th^ National Congress, Turkey, from 3 to 7 October 2021.

### Authors’ ORCIDs

- Mustafa Kayabasi: 0000-0003-2059-0696
- Canan Asli Utine: 0000-0002-4131-2532

### Competing interests

The authors declare that they have no competing interests.

## Supplementary Material

Video of surgical procedure
